# Patterns of Apoptosis and Proliferation throughout the Biennial Reproductive Cycle of Viviparous Female *Typhlonectes compressicauda* (Amphibia, Gymnophiona)

**DOI:** 10.3390/ijms18010016

**Published:** 2016-12-22

**Authors:** Michel Raquet, Claire Brun, Jean-Marie Exbrayat

**Affiliations:** 1 Laboratory of General Biology, Lyon Catholic University, UMRS 449, University of Lyon, 69288 Lyon Cedex 02, France; mraquet@univ-catholyon.fr (M.R.); cbrun@univ-catholyon.fr (C.B.); 2 Laboratory of Reproduction and Comparative Development, Ecole Pratique des Hautes Etudes, Paris Sciences Lettres, 69288 Lyon Cedex 02, France

**Keywords:** apoptosis, proliferation, ovary, oviduct, sexual cycle, amphibian, gymnophiona, *Typhlonectes compressicauda*

## Abstract

*Typhlonectes compressicauda* is an aquatic gymnophionan amphibian living in South America. Its breeding cycle is linked to seasons, characterized by a regular alternation of rainy and dry seasons. During a complex biennial cycle, the female genital tract undergoes a series of alternations of increasing and decreasing, governed by equilibrium of proliferation and apoptotic phenomena. Immunohistochemical methods were used to visualize cell proliferation with the detection of Ki67 antibody, a protein present in proliferative cells; terminal deoxynucleotidyl transferase dUTP nick end labeling (TUNEL) and Apostain were performed to detect apoptotic cells on sections of ovaries and oviducts. In ovaries, both phenomena affect the germinal nests and follicles according to the cycle period. In the oviduct, the balance was in favor of proliferation during preparation for reproduction, and in favor of apoptosis when genital ducts regress. Apoptosis and proliferation are narrowly implicated in the remodeling of the genital tract and they are accompanied by the differentiation of tissues according to the phase of the breeding cycle. These variations permit the capture of oocytes at ovulation, always at the same period, and the parturition after 6–7 months of gestation, at a period in which the newborns live with their mother, protected in burrows in the mud. During the intervening year of sexual inactivity, the female reconstitutes body reserves.

## 1. Introduction

Cellular and organ development is under the equilibrium of three phenomena: proliferation, differentiation and degeneration. These three physiological aspects are particularly highlighted in embryonic development [1,2], and in organs submitted to cyclical variations. Among these organs, the genital tracts of animals can be used such as models to understand the importance of the balance of these three phenomena.

### 1.1. Cell Proliferation and Apoptosis

*Typhlonectes compressicauda* females, as studied in previous works, undergo a biennial sexual Cell proliferation is observed during organogenesis, growth, and reparation after injury or cyclical regression of tissues. This phenomenon has been known for a long time [3,4,5], and corresponds to the different phases of cell cycle (G1, S, G2 and M) [6]. The cell cycle is regulated by various factors including CDKs, cyclin-dependent kinases, some molecules involved in the regulation of cell cycle [7,8], which are submitted to a range of inhibitors, mitogenic factors, and growth factors [9,10].

Apoptosis, first described in 1972 [11], is widely implicated living organisms, particularly in the degeneration and remodeling of tissues [12]. This process is characterized by the fragmentation of DNA provoking a reduction in cell volume and condensation of the cytoplasm. Chromatin condensation begins on the periphery of nucleus before invagination of the nuclear envelope [13,14]. The nucleus and cytoplasm fragmentations are followed by the formation of apoptotic bodies surrounded by fragments of plasma membrane. Apoptotic bodies are quickly phagocyted by macrophages or adjacent cells without any inflammation reaction [11,14,15,16]. Although it is not the only cell destruction process (necrosis and autophagy [17]), apoptosis is a universally widespread and major phenomenon in living systems [18,19]. Indeed, it is observed throughout embryonic development [1,2,20,21]. The development, maintenance or growth of organisms is regulated by the balance between proliferation and apoptosis. This balance is disturbed during the phenomena of carcinogenesis [22,23,24]. Apoptosis also plays an important role in the maturation of the genital organs of animals [25,26,27]. The regulation of apoptosis in follicular development involves gonadotropins and paracrine factors [28]. In the genital tract, apoptosis is involved in constant cell turnover in the oviduct as well as the maintenance of cellular homeostasis [29] and placentation [30,31]. Apoptotic activity is seasonal in the oviduct of vertebrates and represents the key mechanism involved in the remodeling of tissues during the reproductive cycle (in the lizard [32] and the alligator [33]). The removal of cells into the lumen of the oviduct is done only sporadically by apoptosis in primates or cats for example, unlike swine and other mammals [34]. Among amphibians, *Xenopus* is widely used in the study of molecular mechanisms of apoptosis [1,2]. However, studies on the cyclical evolution of the reproductive system among salamanders or tailless amphibians are rare [35,36,37,38,39].

### 1.2. The Species Typhlonectes comzpressicauda

For a long time, our group has studied the variations in reproductive organs in *Typhlonectes compressicauda*, a gymnophionan (or caecilian) amphibian. Gymnophians are burrowing lengthened, limbless and blind animals living in tropical countries. *T. compressicauda* is a secondarily aquatic species living in South America, where it is submitted to marked seasonal alternations. The male cycle is annual [40,41] whilst that of the female is biennial, with a series of well-marked phases of proliferation, differentiation and degeneration of cells in the genital tract [41,42,43,44]. In this manner, *T. compressicauda* represents an ideal model to study the importance of degeneration by apoptosis, with the balance with proliferation and the importance of differentiation.

Moreover, the gymnophionans are amongst the least known amphibians [45,46]; they are interesting for they belong to a homogenous group [47], presenting a lot of particular characteristics [48,49]. In addition, several species of this group are oviparous, viviparous, or presenting a direct development [50,51,52]. Indeed, this amphibian order groups close families, genera and species with different modes of reproduction in which the main cellular physiological phenomena are represented with different modalities. The entire group can be used as such as a model to understand the importance of the distribution of cell phenomena.

The purpose of this present work is to present the importance of apoptosis with proliferation in the variations of the *T. compressicauda* female genital tract throughout the biennial reproductive cycle. Variations of the genital tract, highlighting cell differentiation in *T. compressicauda* were published in several previous studies [40,42,53,54,55,56,57,58].

#### 1.2.1. Ovaries

**Figure 1:**
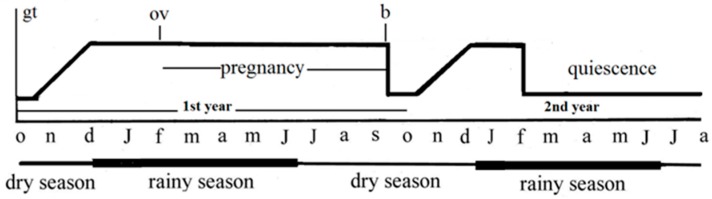
Schematic representation of the biennial sexual cycle in the *Typhlonectes compressicauda* female. The first year is devoted to the pregnancy, and the genital tract is quiescent in the second year. gt: variations of genital tract; ov: ovulation; b: birth. The first letter of each month is given on the abscissa.

*Typhlonectes compressicauda* females, as studied in previous works, undergo a biennial sexual cycle narrowly linked to seasonal variations (Figure 1). At the beginning of cycle, specifically in October of the first year (middle of the dry season), the ovaries of more and more females presented greater numbers vitellogenic oocytes characterized by the presence of yolk platelets and increasing follicles. In December, at the beginning of rainy season, about 100% of females were vitellogenic and ready to breed. Each ovary contained about 20 follicles with vitellogenic oocytes. Ovulation occurred in February, and breeding was observed. After fertilization, the development of embryos was 6–7 months long, and occurred entirely into the oviducts of pregnant females. Parturition was observed in September–October; at this period 6–8 newborn were observed living near their mother, which was burrowing into the mud. After parturition, the ovaries no longer contained any vitellogenic oocytes. The first year of the sexual cycle was thus finished. The second year began with new vitellogenesis. Like the first year, about 20 vitellogenic oocytes were observed in ovaries, but in January–February, ovulation did not occur. Vitellogenic follicles degenerated and became atretic. Finally, the ovaries decreased, and remained inactive until the following October, at which time a new process of vitellogenesis occurred, thereby starting a new biennial cycle. Oogonia and primary oocytes were observed in germinal nests dispersed along each ovary. These nests were more particularly developed from February to April. In non-pregnant females, the atretic follicles showed a continuous evolution. In conclusion, upon reaching a threshold, follicles might continue to develop in two ways: during the breeding period (first year of the cycle), these structures become mature and ovulation occurs; during quiescence (second year of cycle), they degenerate, becoming atretic follicles.

#### 1.2.2. Funnels and Oviducts

The female genital tract of *Typhlonectes compressicauda* is also composed of a pair of funnels and oviducts submitted to seasonal variations.

Each funnel is a groove parallel to the oviduct and to the correspondent ovary. At ovulation, the groove increased, enveloping the ovary. The oocytes were thus laid directly in the funnel and directed to the tubal part of oviduct. During sexual quiescence, the wall was poorly developed, bordered with a single layer of undifferentiated cells, with a large nucleus surrounded by a thin crown of cytoplasm. Just before ovulation, the connective tissue increased, epithelial cells became more voluminous and rounded with a ciliated apical membrane. At ovulation, some crests developed limiting crypts containing cells with protein secretions. After ovulation, the funnel regressed, and became quiescent until the following December. At the second year of the cycle, the funnel regressed at the theoretical time of ovulation.

The anterior part of the oviduct developed at the beginning of cycle. The connective tissue of the wall became rich in cells, vascularized and sent some crests into the lumen. The lumen, first bordered with a simple undifferentiated columnar epithelium, increased, and became bordered with ciliated epithelium. Gland cells developed between the crests. In December–January, oviducts contained acidic mucus. Some cells with sulfated mucus were also found between the ciliated cells. At ovulation, cilia were covered by a thick layer of mucus. Cells lost cilia near oocytes in the lumen. At the beginning of pregnancy, the anterior part regressed. Oocytes that were not fertilized degenerated and yolk platelets became free in the lumen. After pregnancy, the oviduct wall became thin, epithelial cells could be evacuated into the lumen. At the beginning of the second year of the sexual cycle (October), the oviduct again prepared for pregnancy, but it degenerated at the theoretical period of ovulation.

The uterus width increased before ovulation. The wall was bordered with connective tissue forming well vascularized crests. Epithelium was constituted of three cell types. Most cells were equipped with numerous microvilli, rounded ciliated cells were found at the top of crests, some gland cells with acid mucus were observed between the others. At ovulation and at the beginning of pregnancy, cells with microvilli produced long cytoplasm expansions, ciliated cells were covered with acidic mucus produced from the gland cells. Lipid substances covered all cell types. When embryos developed, the uterine wall became distended, secretions of gland cells reduced, and the cells with cytoplasm expansions became degraded. At the end of pregnancy, all epithelial cells degenerated and the connective tissue was directly exposed to the luminal surface. The epithelium of the intra-uterine embryo’s gills was narrowly applied against this connective tissue to give a structure resembling a placenta. After parturition, the uterine lumen became narrow and bordered with an undifferentiated tissue. At the beginning of the second year, a new differentiation of the uterus was observed and degenerated at the theoretical period of ovulation. In the following October, a new biennial cycle started.

Throughout this reproductive cycle, the main organs, such as ovaries and genital ducts, were affected by three fundamental phenomena: cell proliferation, cell differentiation and cell regression. Cell differentiation was studied in several previous works mentioned above [40,42,53,54,55,56,57,58]. In this study, cell proliferation was visualized with the detection of Ki67 antibody, a protein present in proliferative cells and used such as a marker of proliferation used in cancer investigation [59], and apoptosis was visualized using both terminal deoxynucleotidyl transferase dUTP nick end labeling (TUNEL) and “Apostain” method [60].

## 2. Results

### 2.1. Proliferation and Apoptosis in the Ovaries

**Figure 2:**
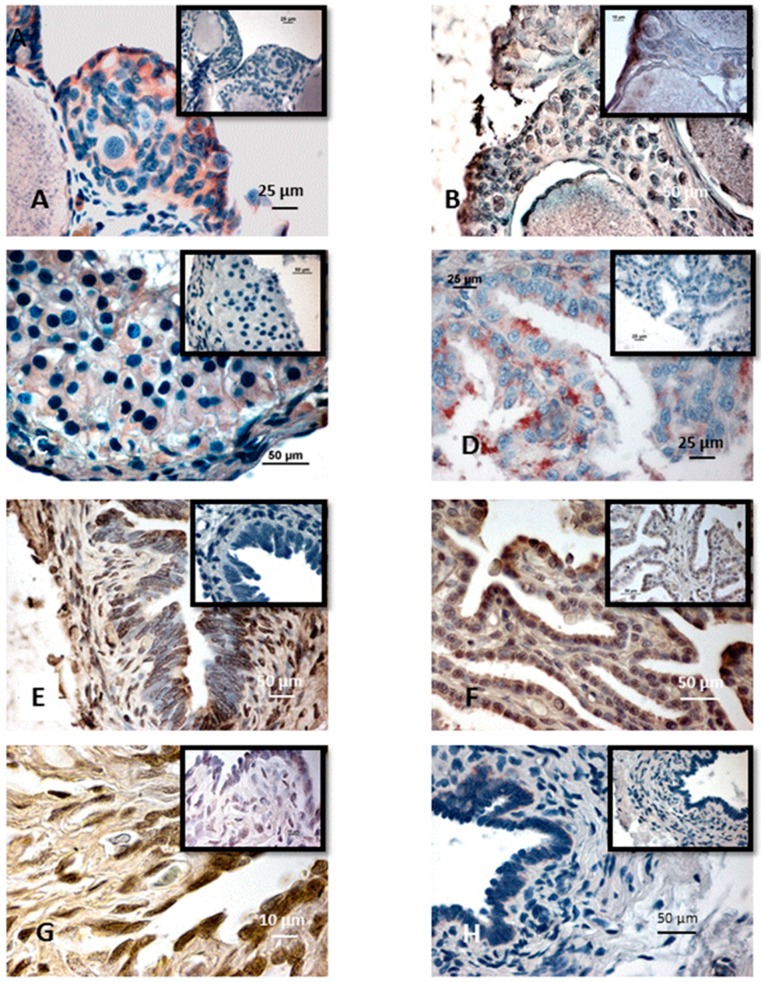
Examples of proliferation or apoptosis in *Typhlonectes compressicauda* genital tract. Inserts show negative controls. (**A**) Cell proliferation detected with Ki67 in a germinal nest of a female during the preparation of reproduction; (**B**) Apoptotic cells detected with TUNEL method in a germinal nest of a female during the preparation of reproduction; (**C**) Cell proliferation detected with Ki67 in a corpus luteum of a pregnant female; (**D**) Cell proliferation detected with Ki67 in the anterior part of oviduct of a no pregnant female; (**E**) Apoptotic cells detected with TUNEL method in the anterior part of the oviduct of a no pregnant female; (**F**) Apoptotic cells detected with Apostain method in the anterior part of the oviduct of a female during the preparation of reproduction; (**G**) Cell proliferation detected with Ki67 in the uterus of a female during the preparation of reproduction; (**H**) Apoptotic cells detected with TUNEL method in the uterus of a pregnant female.

Ki67 immunoreactivity provided a label for the nucleus of some cells (Figure 2A). In negative control, no nuclei were labeled. The proliferation index corresponding to the percentage of labeled nuclei gives a view of mitotic cells. Several apoptotic cells were labeled with both TUNEL and Apostain method (Figure 2B).

#### 2.1.1. Germinal Nests

In this part of the ovary, during the reproductive period, the proliferative indexes were 15.8% for connective cells and 43.3% for oogonia, in pregnant females. During the second year of the cycle, when females were quiescent, these indexes decreased respectively to 0% and 14.6%. Just before the second year of the cycle or after parturition at the onset of a new biennial cycle, proliferative rates increased (13.5% in connective cells and 14.1% in oogonia). The evolution of the number of labeled nuclei by Ki67 detection method was significant through the year (oogonia: *n* = 8, *Pr* = 0.041; connective cells: *n* = 6, *Pr* = 0,003; with *Pr* the probability for differences between the values.to be insignificant; see the details in Material and Methods, Section 4.5).

During the first year of the cycle, 14.3% of connective cells and 8.3% oogonia were labeled with TUNEL method in the ovaries of pregnant females. During the second year, in quiescent females, apoptotic cells affected 10.7% connective cells and no oogonium. At the end of the first year or after parturition, just before the second year of the cycle, 73.3% stromal cells and no oogonium were labeled with TUNEL (Table 1). The Fisher’s test revealed a significant evolution of the number of apoptotic cells between the seasons (oogonia: *n* = 6, *Pr* = 0.013; connective cells: *n* = 4, *Pr* = 0.015).

#### 2.1.2. Growing Follicles

In the growing follicles, the proliferative index of theca was 11.2% during the pregnancy, 2.5% in quiescent females, 23% during the sexual rest, and 24% during the preparation for reproduction; variations between the phases were significant excepted between quiescent females and sexual rest (*n* = 8, *Pr* = 0.002). For granulosa, the proliferative index was 28.2% during the pregnancy, 22% in the quiescent females (second year), 22.5% during the sexual rest, 29.2% after the parturition, and 27.5% during the phase of preparation for reproduction; the increases observed during both the preparation phase and pregnancy were not significant by comparison with the value in quiescent females or in sexual rest, for which they were not different (*n* = 8, *Pr* = 0.106).

In the growing follicles, the percentage of apoptotic theca cells was 14% during the pregnancy, 32.5% during the sexual rest (second year), 13.5% at the end of both the first and second year; these results were significantly different excepted in the quiescent females and females in sexual rest (*n* = 6, *Pr* = 0.024). For the granulosa, 11.3% cells were apoptotic during the pregnancy, 26.2% in the quiescent females, and 23.3% at the end of the years; the decrease observed in pregnancy was significantly different from the other percentages which were not significantly different (*n* = 6, *Pr* = 0.07 around the year, *Pr* = 0.037 between the pregnant females and the others) (Table 2).

#### 2.1.3. Vitellogenic Follicles

The biggest follicles were 1000 to 2000 µm in diameter. The oocyte cytoplasm was filled with numerous yolk platelets. Its voluminous nucleus (10 µm in diameter) was pushed against the plasma membrane. The less developed follicles (young vitellogenic follicles), with a vitellogenic oocyte, were surrounded with a thin vitellin membrane. A striated zone corresponding to microvilli of oocytes underlined the plasma membrane. In the next stages (mature vitellogenic follicles), the follicular cells were much enlarged and contiguous. The granulosa cells became cubical and contiguous. The vitellin membrane became thicker, as well as very loose, and the follicular cells seemed to float within the follicle. Certain of the follicular cells were disposed on two layers, one against the theca, and others against the oocyte.

During the breeding period, in young vitellogenic follicles of pregnant females, the proliferation indexes were 10.8% in theca cells and 37.5% in granulosa cells. In mature vitellogenic follicles, the proliferation index was 17.6% for theca and 40% for granulosa. During the resting period, in quiescent females, theca cells were never labeled with Ki67, and the proliferation indexes for granulosa cells were 17.5% in small vitellogenic follicles and 35% in mature ones. At the end of each year, when ovaries contained only young vitellogenic follicles, the proliferation indexes were 15.8% in theca and 48.5% in granulosa. At the onset of preparation, the proliferation indexes were 14.9% for theca and 55.0% for granulosa (Table 3) (*n =* 6, *Pr* = 0.024 for the theca cells, *Pr* = 0.002 for granulosa). During the breeding period, in young vitellogenic follicles, 10.2% theca cells and 42.2% granulosa cells were apoptotic; in mature follicles, 13.3% theca cells and 43.3% granulosa ones were apoptotic. During sexual rest, young follicles were observed in the ovaries of quiescent females, and apoptotic cells were observed only in the granulosa. In remaining mature follicles, apoptosis affected only 34.5% cells of granulosa. At the end of both the first and second years, only young vitellogenic follicles were observed exhibiting apoptosis in 3.2% of theca cells, and 21.5% granulosa cells. At preparation phase, at the onset of each year of the cycle, apoptosis affected 14.2% theca cells and 33.3% granulosa ones (Table 3). The evolution of apoptotic cells number in the mature follicles was significant only for granulosa (*n =* 8, *Pr* = 0.004) and not for the theca cells (*n =* 6, *Pr* = 0.582). In contrast, the variation of mitotic nuclei number was not significant through the year both in the theca cells and in granulosa (*n =* 6, *Pr* = 0.101 and *Pr* = 0.241, respectively).

#### 2.1.4. Atretic Follicles

The diameter of the biggest atretic follicles was 1200 to 2000 µm. In these follicles, vitellogenic oocytes degenerated with a vacuolated cytoplasm; granulosa cells became hypertrophied and migrated into the oocyte after the lysis of plasma membrane and phagocytosis of yolk platelets. In the smallest atretic follicles (750 µm and less), the cells invaded the smallest oocytes looking like an adipose tissue. Atretic follicles degenerated, giving a mass incorporated into the connective tissue.

During the breeding period, no proliferation index was found on the periphery of atretic follicles after application of Ki67. Inversely, the proliferation index of invasive cells providing from the granulosa was 76.7%. In quiescent females, during the sexual resting period, no cell was proliferating at the periphery but the proliferation index was 61% for internal cells. At the end of both years, the proliferation index of invasive cells was 25.8% but no peripheral cell was labeled with Ki67. When reproduction was prepared, the peripheral cells began to proliferate with a proliferation index of 27.5%; proliferation index of invasive cells was 31.2% at the same period (Table 4). All the variations of annual number were statistically significant (*n =* 6, *Pr* = 0.000 for the peripheric cells, *Pr* = 0.016 for the invasive cells).

During the breeding period, 48.3% peripheral cells were apoptotic, but no invasive cell appeared apoptotic. During the second year, apoptosis affected 36.5% peripheral cells and 15.2% invasive cells. At the end of both of the two years of the cycle, apoptosis affected 8.7% of peripheral cells and 29.1% of invasive cells. During the preparation for reproduction, apoptosis affected 31.3% of peripheral cells and 41.8% of invasive cells (Table 4). The annual variations were significant (*n =* 8, *Pr* = 0.006 for the peripheric cells, and *Pr* = 0.005 for the invasive cells).

#### 2.1.5. Corpora Lutea

After ovulation, follicle cells proliferated and invaded the central cavity left free after ovulation giving a large corpum luteum 1200 to 2000 µm in diameter. These cells were disposed in several layers and at first left a large central cavity filled by an uncharacterized substance. These hypertrophied cells were spherical (30 µm in diameter) or elongated (20 × 40 µm). Blood vessels were first limited to the peripheral theca. The granulosa cells stained with Ki67 (Figure 2C) continued to invade the central cavity. At the periphery, cells originating from the theca infiltrated between the granulosa cells. Blood vessels developed between all of the cells. The central cavity of the corpus luteum was then filled with cells, numbers of them containing vacuoles. At the end of pregnancy, the corpora lutea degenerated, and finally, was reduced to a small mass quickly integrated into the connective tissue.

The proliferation index was 14.7% in the peripheral cells of the corpora lutea, and 35.8% for internal cells. Apoptosis affected 18.5% of peripheral cells and 20.8% of proliferative internal cells (Table 5).

### 2.2. Proliferation and Apoptosis in the Different Parts of Oviducts

#### 2.2.1. Ostium (Funnel)

During the breeding period, proliferation indexes were 0% for connective cells and 31.6% for ciliated cells in pregnant females; during sexual rest, indexes were 7.2% for connective cells and 36.8% for ciliated cells. At the end of each of the two years, proliferation indexes were 38.5% for connective cells and 51.7% for ciliated cells. All these differences were significant (*n =* 8, *Pr* = 0.06 for ciliated cells; *Pr* = 0.003 for connective cells). Apoptosis affected 18.3% of connective cells and 27.6% of ciliated ones during the breeding period, and 22.5% of connective cells, and 18.3% of ciliated cells during sexual rest; at the end of both the first and second year, 38.5% of connective cells and 51.7% of ciliated cells were apoptotic (*n =* 6, *Pr* = 0.004 for ciliated cells and *Pr* = 0.02 for connective tissue) (Table 6).

#### 2.2.2. Oviduct

##### Anterior Part (Tubal Part)

During the breeding period, in pregnant females, the proliferation indexes were 36.7% for connective cells, 21.9% for secretory cells, and 34.3% for ciliated cells. During the year of sexual rest, in quiescent females (Figure 2D), these indexes were low, 3.6% for connective cells, 16.8% for secretory cells and no ciliated cell was proliferating (all these difference were significant except for secretory cells: *n =* 8, *Pr* = 0.362; *Pr* = 0.001 for ciliated cells, *Pr* = 0.002 for connective cells) (Table 7).

During the breeding period, apoptosis affected 30.0% to 38.3% of connective cells, 20.8% to 30.6% of secretory cells, and 20.5% to 30.0% of ciliated cells. In quiescent females, apoptosis (Figure 2E) affected a lesser number of cells: only 13.7% of connective cells, 6.7% of secretory cells, and 5.7% of ciliated cells. At the end of each year, 53.3% of connective cells, 22.5% of secretory cells, and 42.5% of ciliated cells were apoptotic. When the oviduct was prepared for breeding, apoptosis visualized with Apostain method (Figure 2F) or TUNEL affected 20.8% to 21.7% of connective cells, 29.1% to 30.6% of secretory cells, and 25.8% to 27.5% of ciliated cells. All these differences were significant (*n =* 6, *Pr* = 0.008 for ciliated cells; *n =* 6, *Pr* = 0.005 for connective tissue; *n =* 8, *Pr* = 0.039 for secretory cells).

##### Posterior Part (Uterus)

During the breeding period, the nuclei of proliferative cells were stained with Ki67 antibody (Figure 2G). The proliferation indexes were 27.5% for connective cells, 20.4% for secretory cells, and 35.5% for ciliated cells in pregnant females, at all embryonic stages. During sexual rest, these indexes were, respectively, 16.5%, 33.2%, and 0%. At the end of each year, the proliferation indexes were 58.3% for connective cells, 19.6% for secretory cells and 10.7% for ciliated cells. During preparation for reproduction, indexes were, respectively, 12.5%, 36.6%, and 36.8%. Fisher’s test revealed that these differences were significant (*n =* 8, ciliated cells: *Pr* = 0.0001; secretory cells: *Pr* = 0.002 and connective cells: *Pr* = 0.000). During the breeding period, in pregnant females (at all embryonic stages), apoptosis revealed with TUNEL method (Figure 2H), affected 36.6% of connective cells, 10.7% of secretory cells, and 28.3% of ciliated cells. During the year of sexual rest, apoptosis affected 14.3% of connective cells, 18.4% of secretory cells, and 34.1% of ciliated cells. At the end of each year of the cycle, apoptosis affected 43.7% of connective cells, 24.3% of secretory cells, and 33.3% of ciliated cells. No cell was affected by apoptosis. All these annual differences were significant (*n =* 6, *Pr* = 0.024; *Pr* = 0.017 for secretory cells and *Pr* = 0.003 for connective tissue) (Table 8).

## 3. Discussion

### 3.1. Ovaries

The detection of apoptotic cells with TUNEL and Apostain methods gave comparable results when they were both performed.

In germinal nests, the presence of both nuclei labeled with Ki67 indicating a proliferative stage and cells labeled with TUNEL or “Apostain” methods indicating apoptosis showed two different dynamics between connective tissue and germ cells. Oogonia were dividing throughout the year in germinal nests, confirming the hypothesis of a continuous oogenesis [40,54,57]. The proliferation index was maximal during the breeding period and was divided by three in quiescent females during the second year of the cycle and at the end of each year. Inversely, oogonia were little affected by apoptosis. The connective tissue of germinal nests was submitted to cell regeneration at ovulation and reproduction. It was possible apoptosis counterbalanced cell proliferation. Thus, in non-pregnant females in which the connective cells stopped multiplying, apoptosis affected only a small percentage of them. At the end of the first and second year, the percentage of apoptotic cells in connective tissue increased strongly, showing a regression of the somatic part of germinal nests. These results suggest a specific regulation of germinal nests in non-pregnant females with stopping of proliferation, and a true regression of connective tissue during the end of the period or the theoretical period of reproduction.

In both growing and vitellogenic follicles, in pregnant females, theca cells possessed a balance of apoptosis/proliferation which was close to one, suggesting a single cell renewal. In quiescent females, the proliferation index was zero, showing that apoptosis was the main physiological phenomenon and suggesting the regression of theca cells and early follicular atresia. The proliferation index rose after the first and second years, and during preparation for breeding, suggesting renewed folliculogenesis. During breeding, in pregnant females, a proliferation/apoptosis balance was observed in the vitellogenic follicles but not in growing ones for which proliferation dominated. However, in non-pregnant females or at the end of the year, the granulosa of growing follicles was renewed, while in the vitellogenic follicles, proliferation occurred twice as often as apoptosis. This phenomenon suggests a proliferation of granulosa cells and the beginning of atresia in mature follicles.

These interpretations are supported by the analysis of the atretic follicles. When breeding, theca cells did not proliferate, but underwent apoptosis in a third or a half of them, while granulosa cells were rarely apoptotic but presented a high proliferation index. The results also showed a slightly different control of atresia in periods of sexual rest or breeding preparation, with the same balance between apoptosis and proliferation, suggesting a slowing down in the process of atresia.

As in atretic follicles, in the new corpora lutea, thecal cells regenerated and granulosa cells proliferated. This result was consistent with the proliferation and differentiation of granulosa cells that became luteal cells. Apoptosis was particularly active during sexual rest and when atresia occurred, like in other vertebrates [61,62,63,64,65].

### 3.2. Funnels and Oviducts

The detection of apoptotic cells with TUNEL and Apostain methods gave comparable results when both were performed [66,67,68,69]. During the breeding season, proliferation rates were observed close to those of apoptosis throughout the genital tract. These observations would suggest for a turnover of tissues.

The evaluation of the proliferation indexes of ciliated and connective cells was consistent with their trophic role. At gestation, the lamina propria of the uterus appeared rather in cell regression, while the epithelium multiplied in order to ensure its trophic function, as in *Salamandra* [70]. The embryos first fed abrading the epithelium and at the subsequent stages of development, they were narrowly applied against maternal tissues with their enlarged gill forming a placenta-like structure [57]. These observations support the hypothesis of a “mechanical” stimulus, in which embryos eroding the uterine wall would be the cause of the increasing proliferation and also slowing apoptosis. This stimulus could extend the hormonal action of corpora lutea on the tubal epithelium. Only the ciliated cells underwent important cell renewal, and not the secreting cells for which the proliferative index and apoptotic rate were equivalent. This fact was linked to changes in the epithelium of the tubal part [53].

For all tissues in non-pregnant females, connective cells of the funnel and the anterior oviduct, ciliated cells in the uterus and the tubal part of oviduct experienced a proliferation index near zero. Other tissues showed a balance favorable for proliferation: ciliated cells of the funnel, and secretory cells of the oviduct. These phenomena indicated a morphological and functional regression of tissues in non-pregnant females.

The season of sexual rest was characterized by an apoptosis/proliferation balance favorable to apoptosis except for the lamina propria of the uterus in which a turnover appeared. These results were consistent with the morphological regression of the genital tract observed in this season.

During the preparation for breeding, the apoptosis/proliferation balance was reversed in favor of an increasing number of cells. The proliferation rates were significantly higher than those seen in the resting period except for the uterus connective cells. The latter can be interpreted as a slight delay in the remodeling of these tissues, corresponding to the late arrival of embryos in this uterine part. Overall, the increase in cell number appeared to be the primary phenomenon underlying the development of the genital tract after the resting period. The persistence of apoptosis (between 20% and 30%) suggested that this phenomenon was also involved in remodeling of the structures in the genital tract.

Consequently, the proliferation/apoptosis balance appeared to be the underlying cellular phenomenon in modulating the number of cells constituting the female genital tract. It explained the seasonal variations in the thickness of tissue. It also participated in the morphological and functional changes in the genital tract. The remodeling involved the proliferation, apoptosis and differentiation of some cells. These elements support studies conducted in the lizard [32] or in the alligator [33]. These variations were under the hormonal control of both the ovary and pituitary gland, related to the physico-chemical environmental variations [29,71,72,73,74,75,76,77,78]. In non-pregnant females, the genital tract presented an own proliferative and apoptotic profile, consequent to the lack of corpora lutea. These observations suggested a regulation different from that of seasonal sexual rest.

## 4. Materials and Methods

### 4.1. Animals

The animals studied belong to a collection of *Typhlonectes compressicauda* captured in Kaw (French Guyana) throughout the years 1979, 1980, 1981, 1983, and 1987, and stored at the general laboratory of Lyon Catholic University. At this time, no authorization was required to collect animals in the field. The animals (males, pregnant and not pregnant females, and newborn and immature males and females), were fixed in Bouin’s fluid, and stocked in 70% ethanol, or formalin. Genital tracts were dissected from these fixed animals.

This population of *Typhlonectes compressicauda* is submitted to a seasonal alternation characterized by a rainy season from January until June and a dry season from July until December. During the rainy season, the animals live in swamped savannas, where they find an abundant food supply constituted of dead fish, insects and some plants [79]. During this period, males and females can meet, allowing reproduction. During the dry season, the level of water progressively falls. Animals become above water and they burrow in the mud. Newborns are observed at this period, in September or October. Sexual cycles are closely linked to season. The male sexual cycle is yearly, that of the female is biennial. In females, breeding occurs in the first year and the second year is devoted to quiescence. For this work, were studied: two pregnant females, two quiescent females, two female prepared for the breeding and two females after the parturition or at the end of the second year of cycle (it is not possible to distinguish the true state).

### 4.2. Histology and Histochemistry

For the study of cyclical variations, the ovaries and oviducts were stained with classical histological methods: Hemalum–eosine, Masson–Goldner’s trichrome and Romeis’ azan. Periodic acid and Schiff reaction (PAS) was used to visualize mucous secretions, and alcian blue at pH 2.5 was used to visualize acidic carbohydrates.

### 4.3. Detection of Ki67 by Immunohistochemistry

Deparaffinized and rehydrated slides were subjected to antigen retrieval using microwave 800 w, 10 min (Antigen unmasking solution, H-3300, Vector laboratories, Les Ullis, France). After blocking endogenous peroxidase then non-specific binding of antibodies as described in Section 4.4 below, the sections were stained for 1 h at room temperature with anti-Ki67 monoclonal antibodies (1:100; AbcysVP-RM04, Abcys, Marcoussis, France). Species-specific isotype controls were used as negative controls. The slides were then washed in Phosphate Buffered Saline (PBS) and immunoreactions were visualized with a streptavidin-biotin amplification kit using Amino Ethyl Carbazole (AEC) as chromogen (Kit VECTASTAIN Elite ABC kit, Vector laboratories, Peterborough, UK).

### 4.4. Visualization of Apoptosis

Apoptosis was examined using both the TUNEL using the in situ Cell Death Detection Kit POD (Roche Diagnostic, Mannheim, Germany), and when possible, “Apostain” methods used the kit of Abcys, Paris, France.

For TUNEL method, deparaffinized and rehydrated sections were incubated in proteinase K (Sigma-Aldrich Chimie, Lyon, France) for 20 min at 37 °C. In order to block endogenous peroxidases, slides were immersed in 3% H_2_O_2_ before to be transferred to the reaction solution terminal deoxynucleotidyl transferase (TdT) + FITC-labeled dUTP for 60 min at 37 °C. In order to block non-specific binding of antibodies, the sections were immersed in 1% bovine serum albumin before to be incubated with peroxidase-conjugated anti-fluorescein antibodies for 30 min at 30 °C in a wet chamber. Then, sections were submitted to a 3,3′-diaminobenzidine revelation solution with H_2_O_2_, and counterstained with hematoxylin. The slides were mounted with cover slips, using Crystal Mount mounting medium (Sigma, Saint-Quentin-Fallavier, France). The labeled cells appeared brown with light microscopy. Negative controls were performed by omitting TdT; positive controls were performed by using DNAse I at 3000 U·mL^−1^ diluted in a 50 mM Tris/HCl solution for 10 min at room temperature.

For “Apostain” method, the monoclonal antibody F7-26 was directed to apoptotic ssDNA after dsDNA opening with a 56 °C formamide treatment. For that, the deparaffinized and rehydrated sections were first incubated for 20 min in a phosphate buffered saline solution containing saponin (0.2 mg·L^−1^), then they were incubated in proteinase K (20 μg·L^−1^), in 50% formamide (*v*/*v* with distilled H_2_O) for 20 min at 58 °C. In order to block the endogenous peroxidases, they were immersed for 10 min in 3% H_2_O_2_. In order to block nonspecific binding of antibodies, they were immersed 30 min at 37 °C in 1% bovine serum albumin. Slides were then transferred for 30 min to the solution of monoclonal antibody F7-26 in a wet chamber before to be incubated in a secondary horse antibody (anti-mouse IgG-HRP, Impress Vector, Peterborough, UK) at 1:200 for 30 min. Finally, the slides were submitted to 3,3′-diaminobenzidine and H_2_O_2_; they were counterstained with hematoxylin QS, a modified Mayer’s hematoxylin. Preparations were mounted with Crystal Mount. Labeled cells appeared brown-stained. Some negative controls were performed using 1:400 diluted horse serum instead of F7-26 antibody.

### 4.5. Statistical Analysis

Eight females in total were studied. For that, two females were studied for each period (pregnancy, quiescence, after parturition or end of the second year of the cycle, and preparation to breeding); two slides were observed for each organ of female; the number of apoptotic or proliferating positive cells per thirty cells was determined for each cell type.

The Nis element BR software (Nikon, Champigny sur Marne, France) was used. To estimate the significance of differences between obtained means, LSD Fisher’s test was conducted. The probability *Pr* for differences between the means to be insignificant was computed. Differences were considered as significant for *Pr* < 0.05.

## 5. Conclusions

Apoptosis and proliferation are closely implicated in the remodeling of the female genital tract and they are accompanied by the differentiation of tissues according to the specific phase of the breeding cycle. Throughout the first year of the biennial cycle, these variations allow preparation for the capture of oocytes at ovulation, always at the same period, and for parturition after 6–7 months of gestation. At birth, the newborns live with their mother, protected in burrows in the mud. During the year of sexual rest, the genital tract begins to be prepared once again for reproduction, but then regresses and female reserves reconstitute [80]. Apoptosis and proliferation are physiological phenomena giving plasticity to the genital tract and participating in the adaptation of animals to their environment.

## Figures and Tables

**Table 1:** Proliferative indexes (Ki67) and percentages of apoptotic cells (TUNEL/Apostain) in germinal nests in *T. compressicauda*. (A): apoptosis detected with Apostain method. *N*: number of females.

Season	*N*	Tissue	Proliferation	Apoptosis
Reproduction (pregnant females)	2	Connective tissue	15.8 ± 1.2	14.3 ± 3.3
		Oogonia	43.3 ± 9.4	8.3 ± 2.3
Sexual rest (quiescent females)	2	Connective tissue	0	10.7 ± 2.5 (A)
		Oogonia	14.6 ± 2.9	0
After parturition	2	Connective tissue	13.5 ± 2.1	73.1 ± 9.7

**Table 2:** Proliferative indexes (Ki67) and percentages of apoptotic cells (TUNEL/Apostain) in growing follicles in *T. compressicauda* during the year. *N*: number of females.

Season	*N*	Tissue	Proliferation	Apoptosis
Reproduction (pregnant females)	2	Theca	11.2 ± 1.8	14.5 ± 4.9
		Granulosa	28.2 ± 4.6	11.3 ± 5.2
Sexual rest (quiescent females)	2	Theca	2.5 ± 3.5	32.5 ± 3.5
		Granulosa	22.5 ± 3.5	26.2 ± 5.3
After parturition or at the end of the second year	2	Theca	24.0 ± 1.4	13.5 ± 2.1
		Granulosa	29.2 ± 2.0	23.3 ± 4.7
Preparation for reproduction	2/0	Theca	27.5 ± 3.5	
		Granulosa	18.3 ± 2.3	

**Table 3:** Proliferation indexes (Ki67) and percentages of apoptotic cells (TUNEL/Apostain) in *Typhlonectes compressicauda* during the year for young and mature follicles. (A): apoptosis detected with Apostain method. *N*: number of females.

Season	*N*	Tissue	Young Follicles		Mature Follicles	
			Proliferation	Apoptosis	Proliferation	Apoptosis
Reproduction (pregnant females)	2	Theca	13.3 ± 4.7	10.2 ± 3.2	14.7 ± 3.9	13.3 ± 4.7
		Granulosa	36.5 ± 4.7	39.1 ± 8.3	41.2 ± 1.8	43.3 ± 4.7
Sexual rest (quiescent females)	2	Theca	0	25.5 ± 2.5 25 ± 1.4 (A)	0	16.5 ± 2.1 17.5 ± 3.5 (A)
		Granulosa	17.5 ± 3.5	23 ± 4.2 21 ± 2.8 (A)	34.5 ± 2.1	16.5 ± 2
After parturition or at the end of the second year	2/0	Theca	15.8 ± 1.2	3.2 ± 2.4		
		Granulosa	48.5 ± 4.9	21.5 ± 4.9		
Preparation for reproduction	2	Theca			14.9 ± 2.5	14.2 ± 5
		Granulosa			55 ± 7.1	33.3 ± 4.7

**Table 4:** Proliferation indexes (Ki67) and percentages apoptosis indexes (TUNEL/Apostain) in atretic follicles of *Typhlonectes compressicauda* during the year. (A): apoptosis detected with Apostain method. *N*: number of females.

Season	*N*	Tissue	Proliferation	Apoptosis
Reproduction (pregnant females)	2	Peripheral cells	0	49.8 ± 5.3
		Invasive cells	77.5 ± 3.5	0
Sexual rest (quiescent females)	2	Peripheral cells	0	36.5 ± 4.9 (A)
		Invasive cells	61.0 ± 5.6	15.2 ± 4.6 (A)
After parturition or at the end of the second year	2	Peripheral cells	0	8.7 ± 1.8
		Invasive cells	25.8 ± 1.2	29.1 ± 5.9
Preparation for reproduction	2	Peripheral cells	27.5 ± 3.5	31.3 ± 5.2
		Invasive cells	31.2 ± 2.3	40.8 ± 5.9

**Table 5:** Proliferation indexes (Ki67) and percentages of apoptotic cells (TUNEL) in corpora lutea of *Typhlonectes compressicauda*. *N*: number of females.

*N*	Cell Type	Proliferation	Apoptosis
2	Peripheral cells	14.7 ± 0.3	18.5 ± 2.31
	Internal cells	35.8 ± 1.2	20.8 ± 5.9

**Table 6:** Proliferation indexes (Ki67) and percentages of apoptotic cells (TUNEL/Apostain) in funnel of *Typhlonectes compressicauda* during the year. (A): apoptosis detected with Apostain method. *N*: number of females.

Season	*N*	Tissue	Proliferation	Apoptosis
Reproduction (pregnant females)	2	Connective cells	0	18.3 ± 2.3
		Ciliated cells	31.6 ± 2.3	27.6 ± 2.3
Sexual rest (quiescent females)	2	Connective cells	7.2 ± 3.2	22.5 ± 3.2 18.4 ± 2.2 (A)
		Ciliated cells	36.8 ± 4.5	18.3 ± 2.3
After parturition or at the end of the second year	2	Connective cells	18.2 ± 1.9	38.5 ± 4.9
		Ciliated cells	37.5 ± 3.5	51.7 ± 2.5
Preparation for reproduction	2	Connective cells	38.3 ± 2.3	
		Ciliated cells	43.5 ± 2.1	75.0 ± 7.1 (A)

**Table 7:** Proliferation indexes (Ki67) and percentages of apoptotic cells (TUNEL/Apostain) in oviduct of *Typhlonectes compressicauda* during the year. (A): apoptosis detected with Apostain method. *N*: number of females.

Season	*N*	Tissue	Proliferation	Apoptosis
Reproduction (pregnant females)	2	Connective cells	36.7 ± 4.7	30.0 ± 4.7 38.3 ± 2.3 (A)
		Secretory cells	21.9 ± 1.6	20.8 ± 5.9 30.6 ± 3.7 (A)
		Ciliated cells	34.3 ± 8.1	20.5 ± 0.7 30.0 ± 4.7 (A)
Sexual rest (quiescent females)	2	Connective cells	3.6 ± 2.0	13.7 ± 1.8
		Secretory cells	16.8 ± 4.5	6.7 ± 2.5
		Ciliated cells	0	5.7 ± 1.0
After parturition or at the end of the second year	2	Connective cells	37.5 ± 3.5	53.3 ± 4.7
		Secretory cells	14.5 ± 4.3	22.5 ± 3.5
		Ciliated cells	20.8 ± 5.9	42.5 ± 3.5
Preparation for reproduction	2	Connective cells	40.3 ± 6.7	21.7 ± 4.6 20.8 ± 1.2 (A)
		Secretory cells	24.6 ± 2.9	29.1 ± 5.9 30.6 ± 3.7 (A)
		Ciliated cells	66.0 ± 5.7	27.5 ± 3.5 25.8 ± 1.2 (A)

**Table 8:** Proliferation indexes (Ki67) and percentages of apoptotic cells (TUNEL/Apostain) in the uterus of *Typhlonectes compressicauda* during the year. (A): apoptosis detected with Apostain method. *N*: number of females.

Season	*N*	Tissue	Ki67	TUNEL
Reproduction (pregnant females)	2	Connective cells	27.5 ± 3.5	36.6 ± 4.7
		Secretory cells	20.4 ± 0.6	10.7 ± 1.1
		Ciliated cells	35.5 ± 3.5	28.3 ± 2.3
Sexual rest (quiescent females)	2	Connective cells	16.5 ± 2.1	14.3 ± 3.3
		Secretory cells	33.2 ± 2.5	18.4 ± 2.7
		Ciliated cells	0	34.1 ± 1.2
After parturition or at the end of the second year	2	Connective cells	58.3 ± 2.6	43.7 ± 1.7 41.6 ± 2.3 (A)
		Secretory cells	19.6 ± 4.1	24.3 ± 3.3 35.0 ± 7.1 (A)
		Ciliated cells	10.7 ± 1.1	33.3 ± 4.7
Preparation for reproduction	2	Connective cells	12.5 ± 3.5	
		Secretory cells	36.6 ± 4.7	
		Ciliated cells	36.8 ± 4.5	
